# Comparison of hemodynamic effects of propofol or alfaxalone during induction in dogs

**DOI:** 10.3389/fvets.2024.1442670

**Published:** 2024-09-11

**Authors:** Diego Sarotti, Paolo Franci, Stefano Oricco, Roberto Rabozzi, Elena Lardone

**Affiliations:** ^1^Centro Veterinario Fossanese, Cuneo, Italy; ^2^Department of Veterinary Science, University of Turin, Grugliasco, Italy; ^3^Centro Veterinario Imperiese, Imperia, Italy; ^4^Policlinico Veterinario Roma Sud, Rome, Italy

**Keywords:** alfaxalone, propofol, hemodynamic variation, dog, anesthesia

## Abstract

This randomized prospective clinical study aimed to compare the hemodynamic effects of propofol and alfaxalone for the induction of anesthesia in dogs. Thirty-one healthy dogs undergoing various procedures in a private referral center were premedicated with intramuscular acepromazine (0.015 mg/kg) and methadone (0.15 mg/kg). They then received 5 mg/kg of propofol over 30 s for induction, followed by a maintenance dose of 25 mg/kg/h (Group P), or 2 mg/kg of alfaxalone over 30 s for induction, followed by a continuous rate infusion of 10 mg/kg/h (Group A). Heart rate (HR), mean arterial pressure (MAP), and the velocity time integral (VTI) of the aortic blood flow using transthoracic echocardiography were measured before anesthetic induction and every 15 s for 180 s. Dogs not adequately anaesthetized for intubation were excluded from the hemodynamic evaluation. Events of hypotension (any MAP value lower than 60 mmHg) were also recorded. Statistical analyses utilized ANOVA for repeated measures, two-way repeated measures ANOVA, paired t-tests, or Wilcoxon signed rank-test as appropriate. Significance was set at *p* < 0.05. Two dogs in Group P (2/14) and 3 in Group A (3/17) were excluded from the study because the anesthesia plane was too light to allow intubation. Treatment P resulted in a significant decrease in MAP between 45 and 75 s during the induction period, with no significant variation in HR, VTI, and VTI*HR. In treatment A, HR increases between 60 and 105 s, VTI decreases at 150–180 s. Analysis between groups did not show any difference in MAP (*p* = 0.12), HR (*p* = 0.10), VTI (*p* = 0.22) and VTI*HR (*p* = 0.74). During induction, hypotension was detected in 3/12 (25%) dogs in Group P and 1/14 (8%) in Group A. In healthy premedicated dogs, propofol and alfaxalone induction produce similar hemodynamic variations. Propofol induction results in a short-term reduction in MAP, whereas alfaxalone induction preserves MAP and cardiac output by significantly increasing heart rate.

## Introduction

Since alfaxalone has been reformulated in 2-hydroxypropyl-b-cyclodextrin, a synthetic carbohydrate molecule not associated with allergic reactions, interest in its use in dogs and cats is growing. With propofol, alfaxalone is the most widely used injectable anesthetic agent in veterinary practice in dogs. Their properties have been extensively studied in the scientific literature, including their pharmacokinetics with or without other drugs, factors affecting the quality of induction and recovery, effects on laryngeal movement, use in cesarean section, and ocular and respiratory effects (1–7). However, there is currently a lack of research on the hemodynamic effects of alfaxalone in the first minutes of induction and its comparison with propofol.

During induction, the major cardiovascular changes would be expected to occur within the first few minutes of induction, transitioning from full consciousness to anesthesia. Cattai et al. (8) found that the most pronounced haemodynamic depression (T-peak) occurred approximately 1 min [55 (50–60) sec] after the start of propofol administration in healthy premedicated dogs.

Several studies in humans and animals have focused their attention on the first minutes after anesthetics administration to characterize the most relevant hemodynamic changes (9–11). Beat-to-beat monitoring of cardiac stroke volume can be considered crucial monitoring to overcome the difficulties of evaluating the rapid haemodynamic changes during induction of general anesthesia. In this context, the velocity time integral (VTI) obtained by transthoracic echocardiography can be used as a surrogate for stroke volume during induction of anesthesia (8, 12–14). The aim of our study was to compare the hemodynamic changes over 180 s, including VTI, heart rate (HR), and mean arterial pressure (MAP), caused by intravenous administration of propofol and alfaxalone over a period of 30 s in healthy premedicated dogs undergoing various procedures. We hypothesized that induction with alfaxalone would result in minimal cardiovascular depression, comparable to or lower than propofol administration.

## Materials and methods

The study was approved by the Ethics Committee of the University of Turin (Prot. N.70/10/01/2020).

### Animals

Eligible for the study were dogs admitted to the Centro Veterinario Fossanese for various scheduled procedures. All animals underwent a physical examination and blood analysis. Dogs were included in the study if they met the following criteria: consent given by the owner, ASA physical status classification I, and age over 1 year.

### Study protocol

All dogs were premedicated intramuscularly with acepromazine 0.015 mg/kg (Fatro S.p.A., Ozzano dell’Emilia, Italy) and methadone 0.15 mg/kg (Dechra, Bladel, Netherlands). After 30 min, two catheters were aseptically inserted, one into a cephalic vein and one into a dorsal pedal artery. Respiratory and cardiovascular variables were monitored using a multiparameter monitor (Datex Ohmeda AS/3, GE Healthcare). Dogs were placed in the right lateral recumbent position and continuous invasive blood pressure monitoring was started. Hair was clipped over the xiphoid area. Transthoracic echocardiography was used to measure aortic VTI using a phased array probe (Samsung HS50, PA3-8B). Two-dimensional cine loops and Doppler tracings were obtained from the subcostal view and ECG trace recording. Images were recorded for off-line analysis.

Dogs were randomly assigned to receive induction with propofol 10 mg/mL (Propofol; Esteve, Italy) (group P) or alfaxalone (Alfaxan; Vetoquinol, France) diluted to 5 mg/mL with 0.9% sodium chloride (group A). The inductors were mounted on a syringe driver (Graseby 3,500, Smiths Medical, England) programmed to deliver propofol intravenously at a rate of 5 mg/kg over 30 s, followed by a continuous infusion of 25 mg/kg/h, or alfaxalone at a rate of 2 mg/kg over 30 s, followed by a continuous infusion of 10 mg/kg/h. Every dog has been preoxygenated before induction for 3 min.

One minute after the start of the induction, each dog was assessed for the first time for the possibility of endotracheal intubation. Signs that intubation was feasible included a depressed eyelid reflex, rostromedial eye rotation, reduced jaw tone and lack of tongue retraction. Once these signs were observed, endotracheal intubation was performed, and the dog was connected to a circuit breathing system for 100% oxygen delivery. All dogs were ventilated with a mechanical ventilator to provide positive pressure control with a paw of 10 cm H2O (Avance CS, GE Healthcare, Chalfont St Giles, UK). Dogs that could not be intubated within 2 min due to light anesthesia were excluded from haemodynamic evaluation.

Aortic flow, heart rate and MAP were recorded before induction and every 15 s for 180 s after the start of the induction bolus (time zero). Heart rate and MAP were recorded using commercial software (Monitor Software version 6.1, University of Hong Kong). Aortic velocity time integral was obtained by averaging 6 consecutive measurements from digital still images. Only high-quality images were used for data analysis.

Echocardiographic measurements were performed by an experienced operator (SO) and all data collected were analyzed off-line by a blinded investigator (DS).

VTI multiplied by HR (VTI*HR) was reported as a surrogate for cardiac output. Events of bradycardia (HR < 60 beats/min) and hypotension (any MAP less than 60 mmHg) were also recorded.

### Statistical analysis

The G*Power Version 3.1 (Heinrich Heine, Universitat Dusseldorf) was used for the sample size calculation, and the following were used for the calculation: Test family = F, Statistical test = ANOVA (Repeated measures between factors), Effect size *f* = 0.20, alpha error probability = 0.05, 1-beta error probability = 0.80, Number of groups = 2, number of measurements = 4, correlation among repeated measures = 0.3. The total sample size was calculated as 26 between subjects. The statistical software used in statistical analysis was URL https://datatab.net. The Kolmogorov–Smirnov test was used to assess the normality of distribution. Heart rate, MAP, VTI, and HR*VTI between-group were analyzed with two-way ANOVA, repeated measures, with Bonferroni post-hoc correction. The same parameters within-group with one-way ANOVA, repeated measures, were performed between baseline and post-induction values, using the Bonferroni post-hoc test. Paired t-tests or Wilcoxon signed rank-tests were used as relevant. Results are presented as mean ± standard deviation (SD) or median (range), as appropriate. Significance was set at *p* < 0.05.

## Results

The study excluded two dogs from Group P and three from Group A because their level of anesthesia was not deep enough to allow intubation. On the other hand, 12 dogs from Group P and 14 dogs from Group A met the criteria and were included in the analysis. The two groups were not different in age, weight, and ASA status (*p* > 0.05) (Table 1). The dogs were a mix of breeds, including 9 Mixed breed, 1 Labrador Retriever, 1 Jack Russel Terrier, 1 Lagotto Romagnolo in Group P and 10 Mixed breed, 2 German Shepherd and 2 Shih-tzu in Group A.

**Table 1:** Demographic data in Group P (Propofol) and Group A (Alfaxalone).

	Group P (12)	Group A (14)	*p*-value
Age (years)	4.5 (1–15)	4 (1–15)	0.71
Weight (kg)	17.5 (3–30)	11 (8–32)	0.78
Sex	5 Male and 7 Female	6 Male and 8 Female	1

The median intubation time after the start of the infusion was 65 (50–110) seconds in Group P and 77 (58–128) in Group A, respectively (*p =* 0.01). During observation period in Group P, 3 out of 12 (25%) dogs experienced hypotensive events, while in Group A, 1 out of 14 (7%) dogs did (*p* = 0.3). No bradycardia events were recorded.

The hemodynamic values before induction (T_0_), including MAP, HR, VTI, and VTI*HR, did not differ between groups (*p* > 0.05).

**Figure 1:**
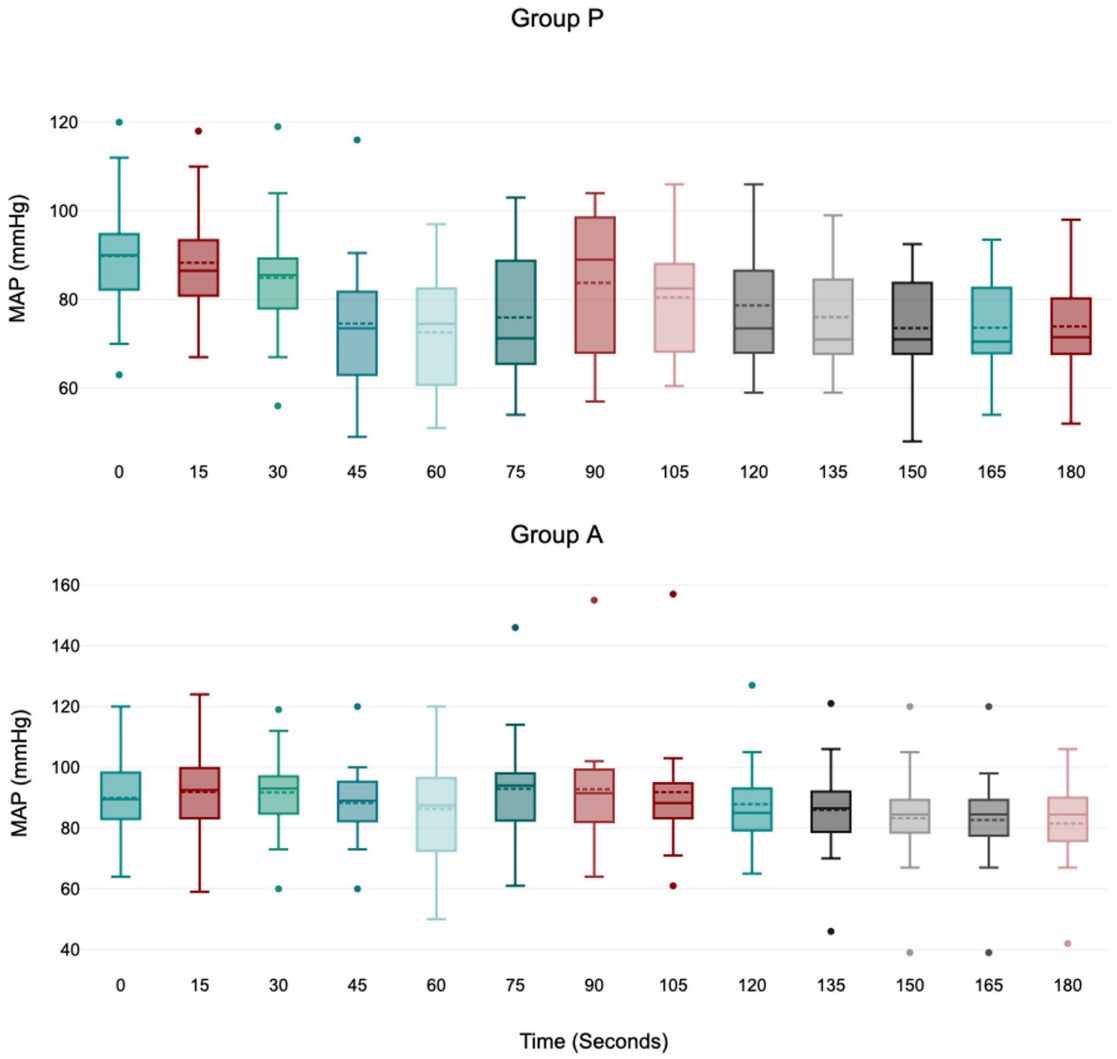
Values of MAP (mean arterial blood pressure) during induction in Group P (12 dogs) and Group A (14 dogs). Within Group P, a significant decrease from baseline in MAP was detected at 45–60–75 s (*p* = 0.01; *p* = 0.01; *p* = 0.04). Within Group A, there is no difference in MAP. The central box represents the values from the lower to upper quartile, the middle solid line the median, the spotted line the mean, spots the outliers, and whiskers the range values.

**Figure 2 fig2:**
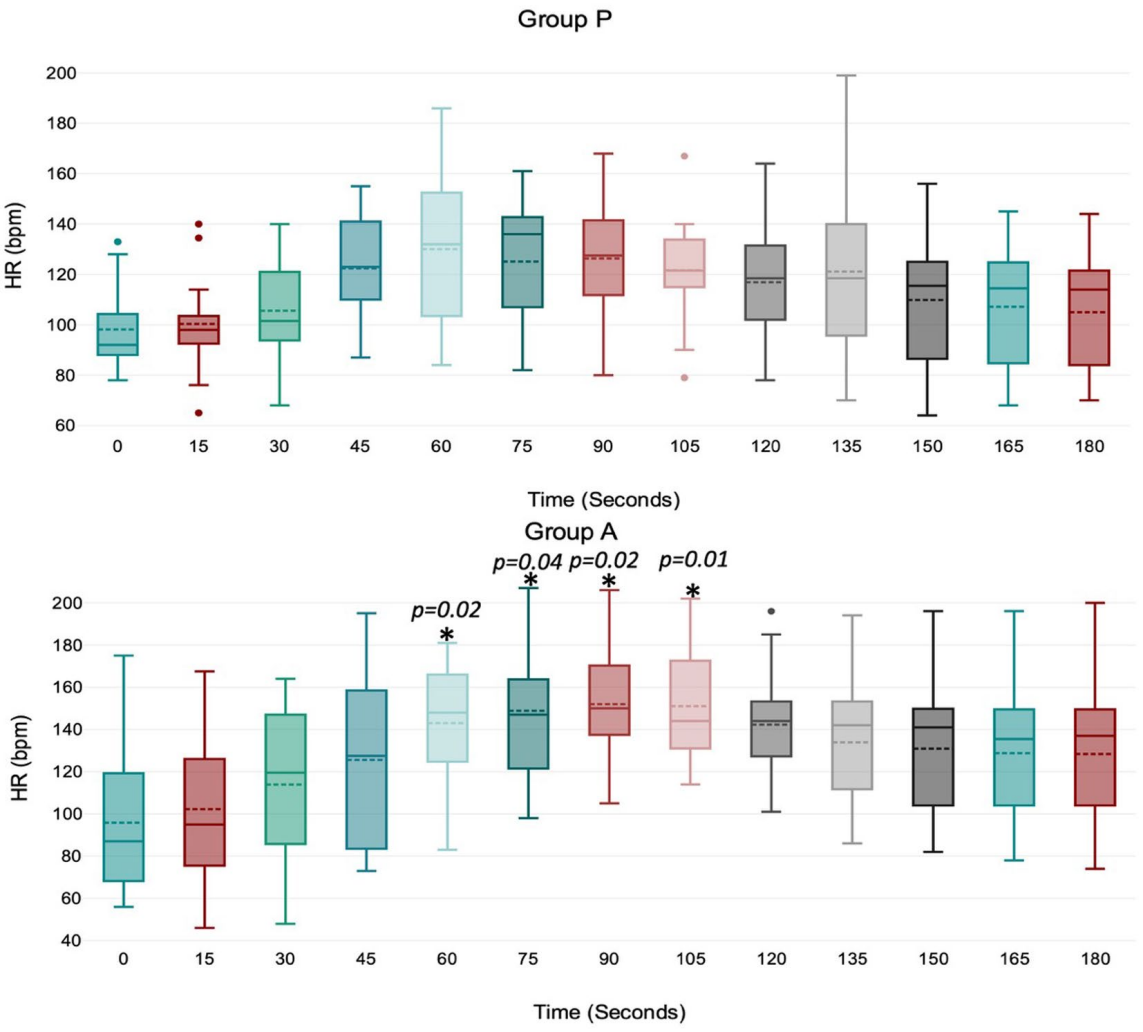
Values of HR (heart rate) during induction in Group P (12 dogs) and Group A (14 dogs). Within Group P, there is no difference in HR. Within Group A, a significant increase from baseline in HR was detected at 60–75–90–105 s (*p* = 0.02; *p* = 0.04; *p* = 0.02; *p* = 0.01). The central box represents the values from the lower to upper quartile, the middle solid line the median, the spotted line the mean, spots the outliers, and whiskers the range values.

**Figure 3 fig3:**
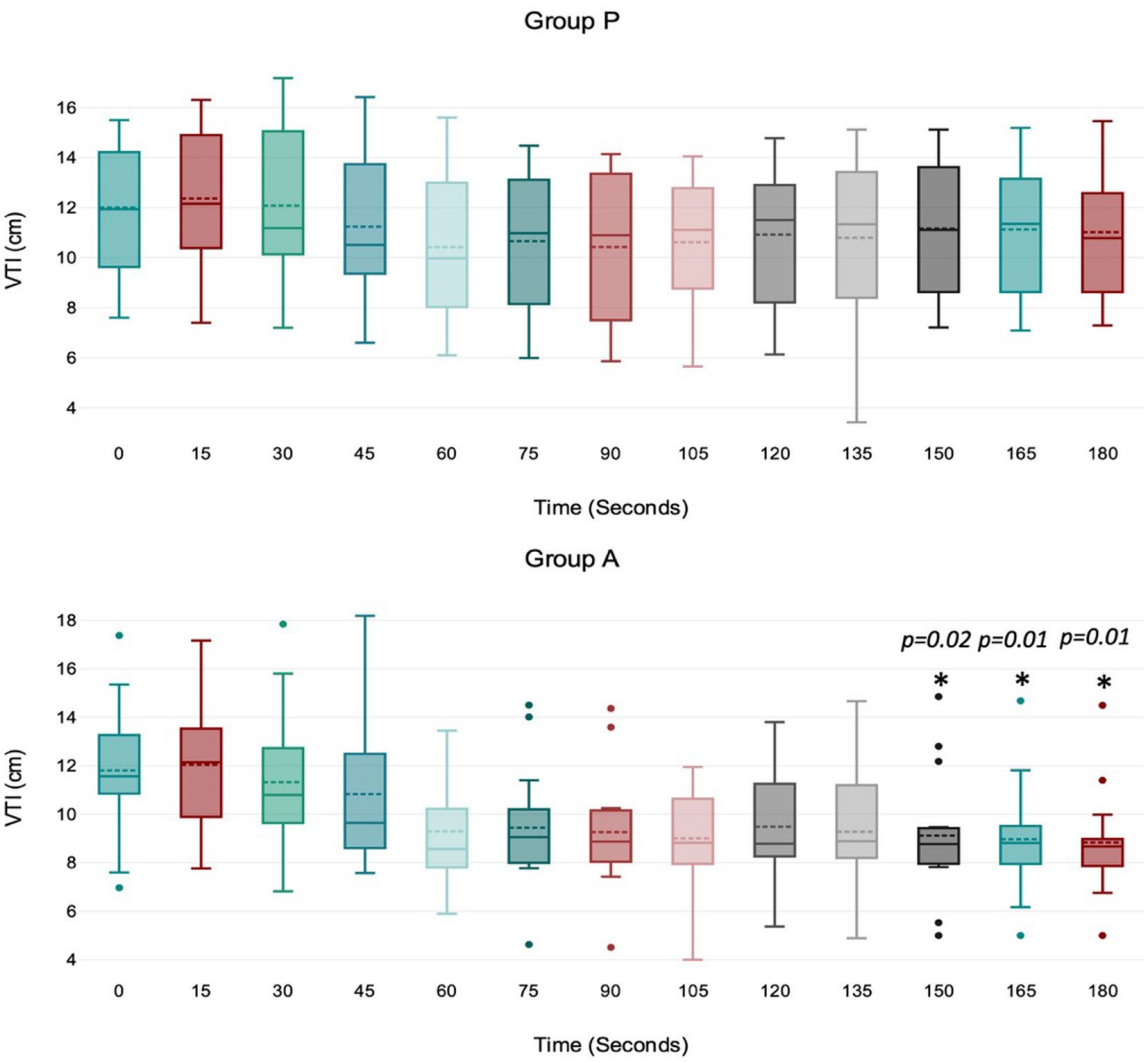
Values of velocity time integral (VTI) of the aortic blood during induction in Group P (12 dogs) and Group A (14 dogs). Within Group P, there is no difference in VTI. Within Group A, a significant drop from baseline in VTI was detected at 150, 165, and 180 s (*p* = 0.02, *p* = 0.01, *p* = 0.01). The central box represents the values from the lower to upper quartile, the middle solid line the median, the spotted line the mean, spots the outliers, and whiskers the range values.

**Figure 4:**
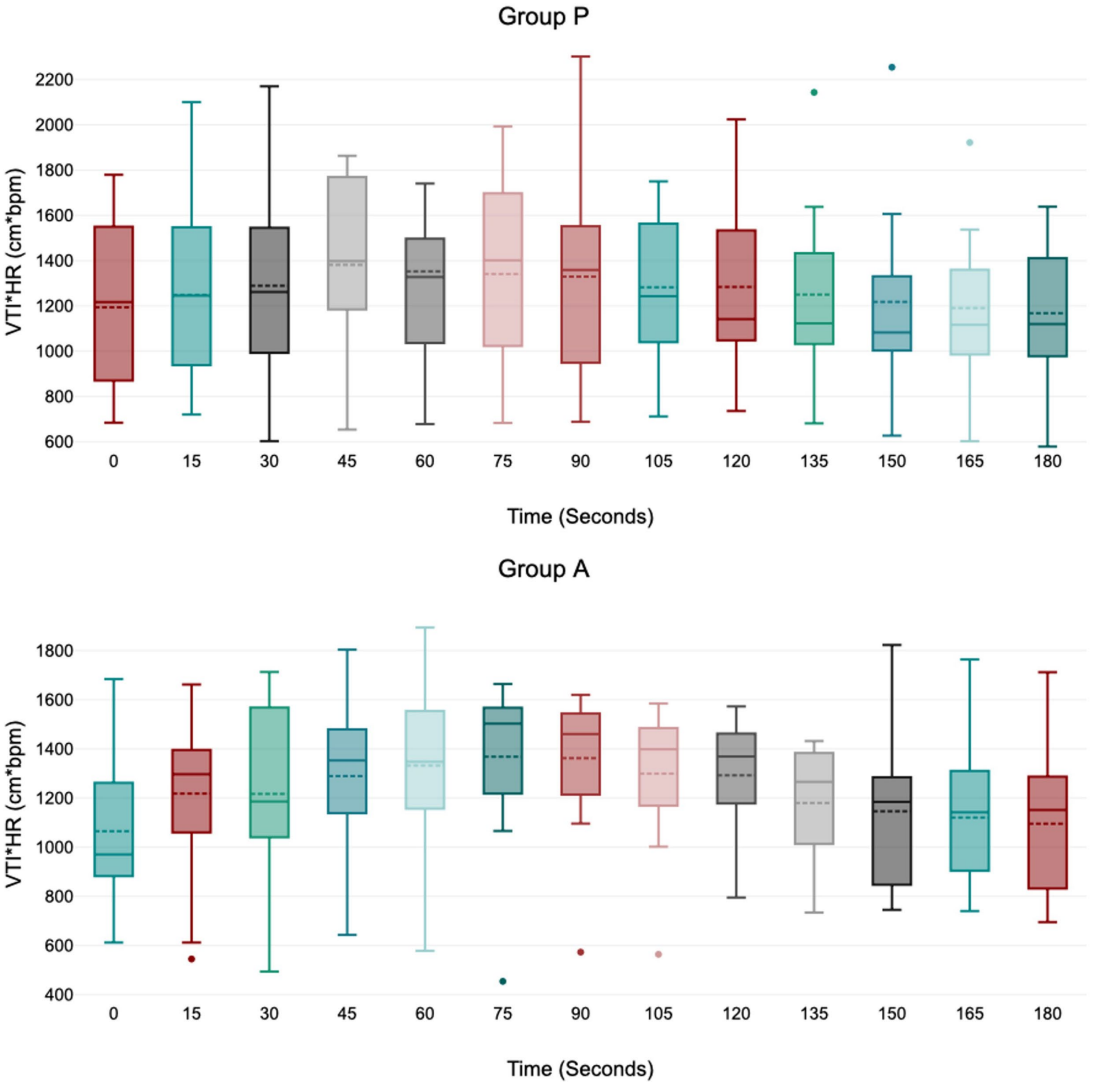
Values of velocity time integral of the aortic blood multiplied by heart rate (VTI*HR) during induction in Group P (12 dogs) and Group A (14 dogs). Within Group P and Group A, there is no difference in VTI*HR. At 45 s in group A VTI*HR is borderline with statistically significance (*p* = 0.05). The central box represents the values from the lower to upper quartile, the middle solid line the median, the spotted line the mean, spots the outliers, and whiskers the range values.

Treatment P led to a significant decrease in MAP during induction between 45 and 75 s (Figure 1), with no significant variation in HR, VTI, and VTI*HR (Figures 2–4).

Treatment A did not result in significant MAP and VTI*HR variation during the observation period (Figures 1,2 [Fig fig3] 4). Heart rate increased between 60 and 105 s (Figure 2), and VTI showed a decrease at 150–180 s (Figure 3). Table 2 summarizes the differences in haemodynamic values within groups compared to baseline and shows the median intubation time in both groups.

**Table 2:** Differences of hemodynamic values within groups compared to baseline and median intubation time in Group P and Group A.

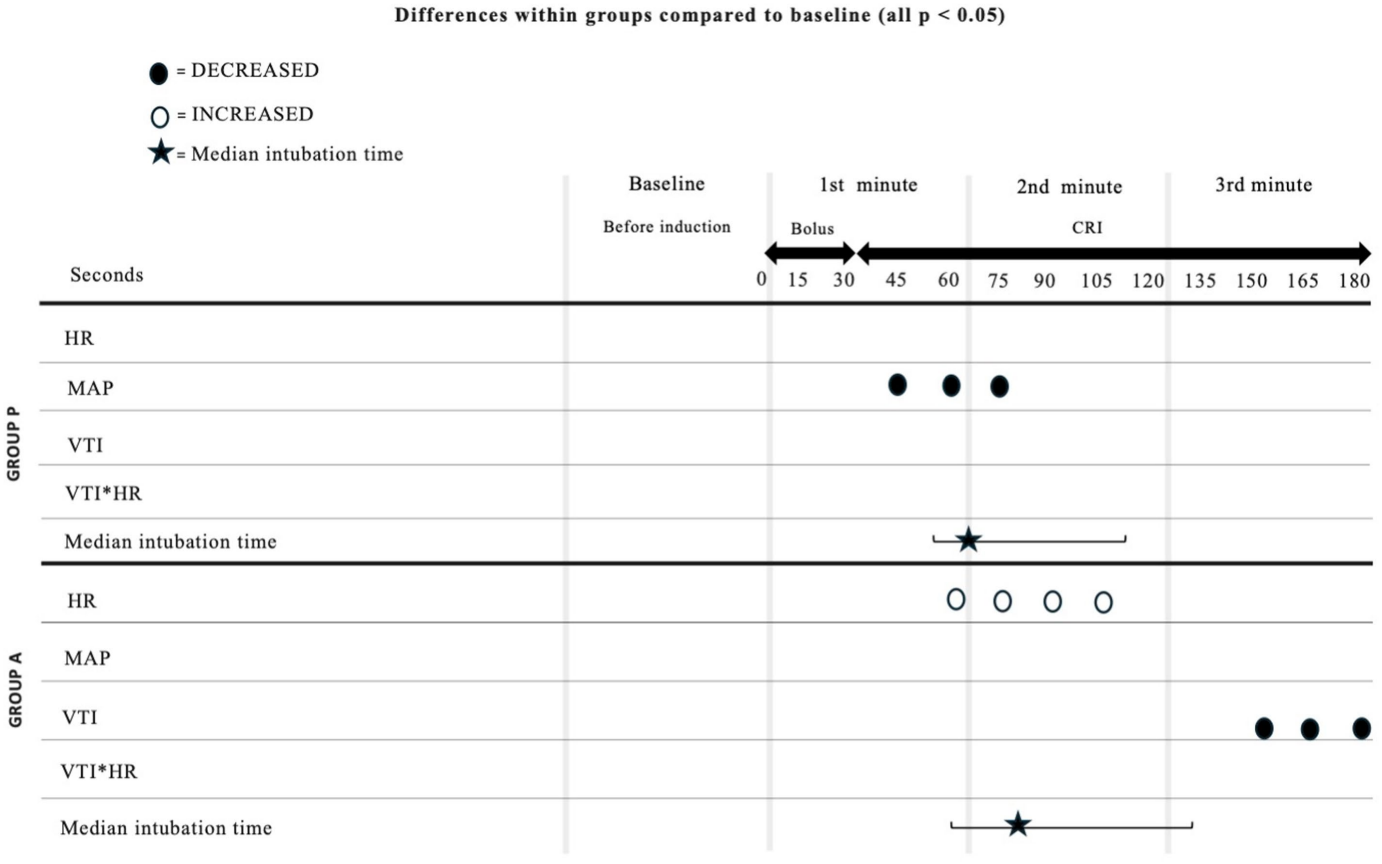

**Figure 5:**
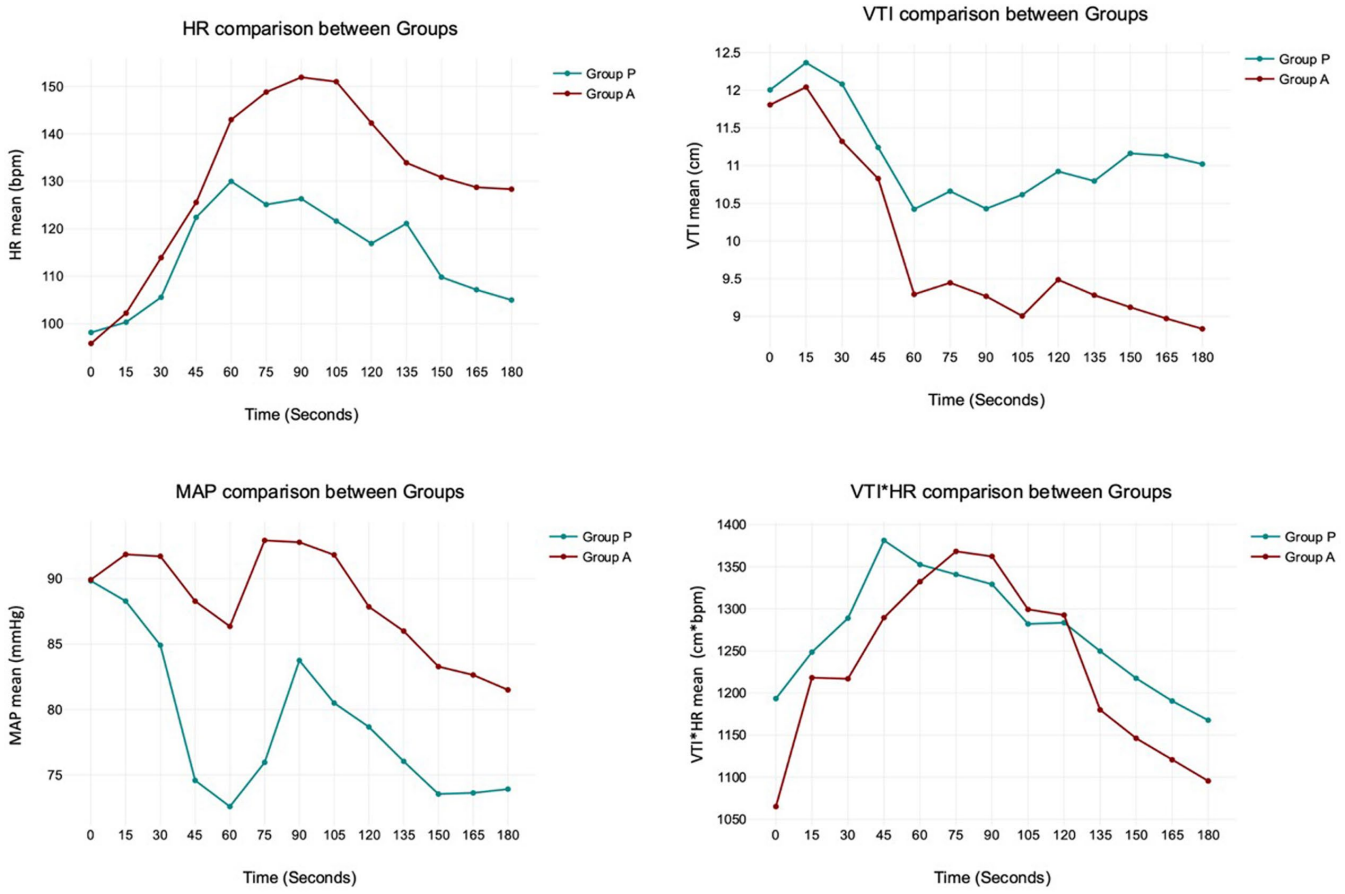
The trend of the mean value of MAP, HR, VTI and VTI*HR in both Groups. The analysis between Group P and A does not show any difference during the overall induction time in MAP (*p* = 0.12), HR (*p* = 0.10), VTI (*p* = 0.22), and VTI*HR (*p* = 0.74).

The analysis between groups did not show any difference during the overall induction time in MAP (*p* = 0.12), HR (*p* = 0.10), VTI (*p* = 0.22) and VTI*HR (*p* = 0.74). Figure 5 shows the trend of the mean value of MAP, HR, VTI and VTI*HR in both groups.

## Discussion

Propofol and alfaxalone induced comparable haemodynamic changes during anesthesia induction. This study did not identify statistically significant differences in MAP, HR, VTI and VTI*HR between groups within the first 180 s following the administration of these drugs in healthy dogs.

We observed some unique hemodynamic characteristics associated with propofol and alfaxalone. Alfaxalone caused a noticeable increase in HR between 60 and 105 s, peaking at 90 s. This chronotropic effect, likely reflecting a baroreceptor response, has been observed in several other studies (15–18). A more pronounced sympathetic response to vasodilation occurred with alfaxalone compared to propofol, possibly due to better preservation of the baroreceptor reflex. To date, we cannot exclude the possibility of alfaxalone affecting HR even through a different mechanism. An experimental study in dogs (19) showed that vagal activity was significantly reduced after administering a mixture of alfaxalone and alphadolone (Althesin), almost to zero, with only a slight increase in the sympathetic discharges. Unfortunately, isolating the effects of baroreceptor response from blood pressure decrease, as well as the influence of the autonomic nervous system in a clinical study model, is challenging and further studies are required to confirm this hypothesis. The increase in HR during alfaxalone use could be interpreted as either positive or negative. In healthy dogs of our study, the positive chronotropic response contributed to maintaining stable values of arterial pressure and cardiac output; however, the same effect could be contraindicated in patients in whom an excessive increase in HR is not tolerated, such as in dogs with myocardial disease or altered hemodynamics (16, 20). However, it is not known whether the chronotropic effect occurs in diseased animals.

In Group P, the HR increase was not statistically significant; however we cannot exclude that a larger sample size might reveal a chronotropic effect. Moreover, the administration of the premedication drugs may have influenced the chronotropic response after propofol induction in dogs. Studies have reported an increase in HR in dogs premedicated with acepromazine and methadone (8, 18), a decrease in HR in dogs premedicated with fentanyl (16) and no change in HR in dogs premedicated with acepromazine-pethidine or not premedicated at all (17, 21).

The mean arterial pressure decreased progressively, reaching its minimum value at 60 s in both groups. However, the drop in MAP only had statistical significance in group P, between 45 and 75 s. The study by Cattai et al. (8) suggested a possible explanation of a direct drug effect on the cardiovascular system due to the peak plasmatic concentration of propofol, which is consistent with the cardiovascular T-peak observed around 60 s after the administration. Furthermore, 3 dogs in Group P developed moderate and transitory hypotension (MAP>50 mmHg for <45 s), whereas only one in Group A did. We cannot exclude the possibility that the incidence of hypotension may have been statistically significant with a larger sample size.

The product VTI*HR, a surrogate for cardiac output, remained constant in both groups except for a probable but statistically borderline increase in group A at 45 s (*p =* 0.05). This data agreed with Rodríguez et al. (20), which reported a cardiac index (measured with a PiCCO monitor) increased at 60 s following alfaxalone induction. This effect was most likely due to a transient increase in HR and a slight decrease in peripheral vascular resistance (decreased afterload) secondary to peripheral vasodilation. The ability of the alfaxalone to maintain or slightly increase the patient’s cardiac output during induction of anesthesia was confirmed by Muir et al. (15), after an IV administration of 2 mg/kg. Unfortunately, Muir’s study provided no hemodynamic data within the first minute post-administration. In Group P, an increase of VTI*HR did not reach statistical significance. This finding agreed with a previous paper (8) that had already studied the cardiac output in 8 dogs after an induction with propofol, showing no difference in this value.

Therefore, both Group A and Group P maintained stable VTI*HR values despite an increase in HR in Group A. This suggested a potential increase in myocardial oxygen consumption in the alfaxalone group due to the elevated HR. Increasing cardiac output, whether by augmenting stroke volume or HR, could lead to varying degrees of myocardial oxygen demand. While this variation may be inconsequential in healthy patients, it becomes particularly relevant in dogs with hemodynamic instability.

The aortic VTI decreased between 150 and 180 s in the alfaxalone group. This finding is likely due to the high heart rate in this group. However, other factors may have contributed to this trend. Firstly, the decrease in stroke volume could be associated with a negative inotropic effect, as indicated by a reduction in MAP. Secondly, the rapid bolus injection of alfaxalone may have caused venous vasodilation, leading to reduced stroke volume due to decreased venous return and preload. Additionally, the recovery of afterload following its reduction during the rapid induction phase with alfaxalone could also be a contributing factor. Nevertheless, a decrease in MAP would not typically be expected in this scenario (1).

Although the populations of the two groups did not have statistically different baseline hemodynamic values, a potential criticism of the current study was the wide range of values before treatments. We could not exclude the fact that the patient’s hemodynamic condition could influence the cardiovascular response. Furthermore, the sample size could also impact certain results, and we could not disregard potentially diverse trends in a larger population where individual differences may be less pronounced.

The bolus doses of propofol and alfaxalone were chosen based on the literature (1, 22). The doses and infusion rates used in this study were a compromise between the safety of the subjects enrolled and the attempt to demonstrate the cardiovascular effects of the two drugs by rapid administration, which has been shown to be more likely to cause marked cardiovascular depression (23). In this perspective, the exclusion of dogs from the study due to inadequate anesthesia was a consequence of this compromise. Nevertheless, several factors affect the dose requirement, such as premedication, body condition score, time of bolus administration, age, and inductor dilution (24–30). The relatively high infusion rate at the end of the induction bolus for both drugs is justified by the high capacity to distribute a liposoluble anesthetic from the central compartment to the fast peripheral compartment during the first part of anesthesia (30). Therefore, to maintain a constant blood drug concentration after induction of anesthesia, the drug infusion rate should be kept rather high. Subsequently, the infusion rate can be reduced to avoid overdosing. The doses used in our study resulted in an appropriate level of hypnosis for most of the patients and, above all, the intubation failure rate (around 20%) was similar in both groups.

Moreover, the median intubation time after the start of the infusion was statistically different (*p =* 0.01) between Group P, with 65 (50–110) seconds, and Group A, with 77 (58–128), respectively. The data suggested that the alfaxalone group was given a lower dose of the anesthetic drug than the propofol group, which may have resulted in minor hemodynamic effects on the patients. However, we could only consider this as a speculation due to the low number of patients and the absence of other groups with different dosages or infusion rates.

In conclusion, the choice between propofol or alfaxalone is unlikely to have a significant clinical impact in healthy dogs. Both agents would seem to have a suitable cardiovascular pharmacodynamic profile for use as an anesthetic induction agent in healthy premedicated dogs: alfaxalone better preserved arterial blood pressure than propofol by significantly increasing heart rate.

## Data Availability

The raw data supporting the conclusions of this article will be made available by the authors, without undue reservation.
